# Reduced Colonic Mucosal Injury in 2,3,7,8-Tetrachlorodibenzo-*p*-Dioxin Poly ADP-Ribose Polymerase (TIPARP/PARP7)-Deficient Mice

**DOI:** 10.3390/ijms23020920

**Published:** 2022-01-15

**Authors:** David Hutin, Karoline Alvik Hagen, Peng Shao, Kim Sugamori, Denis M. Grant, Jason Matthews

**Affiliations:** 1 Department of Pharmacology and Toxicology, University of Toronto, Toronto, ON M5S1A8, Canada; dhutin1@gmail.com (D.H.); peng.shao@mail.utoronto.ca (P.S.); ks.sugamori@utoronto.ca (K.S.); denis.grant@utoronto.ca (D.M.G.); 2 Department of Nutrition, Institute of Basic Medical Sciences, University of Oslo, 0317 Oslo, Norway; k.a.hagen@medisin.uio.no

**Keywords:** poly-ADP-ribose polymerase 7 (PARP7), aryl hydrocarbon receptor, colitis, mono-ADP-ribosylation

## Abstract

Poly-ADP-ribose polymerases (PARPs) are important regulators of the immune system, including TCDD-inducible poly-ADP-ribose polymerase (TIPARP), also known as poly-ADP-ribose polymerase 7 (PARP7). PARP7 negatively regulates aryl hydrocarbon receptor (AHR) and type I interferon (IFN-I) signaling, both of which have been implicated in intestinal homeostasis and immunity. Since the loss of PARP7 expression increases AHR and IFN-I signaling, we used a murine dextran sulfate sodium (DSS)-induced colitis model to investigate the effect of PARP7 loss on DSS-induced intestinal inflammation. DSS-exposed *Parp7^−/−^* mice had less body weight loss, lower disease index scores, and reduced expression of several inflammation genes, including interleukin *IL-6*, C-x-c motif chemokine ligand 1 (*Cxcl1*), and lipocalin-2, when compared with wild-type mice. However, no significant difference was observed between genotypes in the colonic expression of the AHR target gene cytochrome P450 1A1 (*Cyp1a1*). Moreover, no significant differences in microbial composition were observed between the genotypes. Our findings demonstrate that the absence of PARP7 protein results in an impaired immune response to colonic inflammation and suggests that PARP7 may participate in the recruitment of immune cells to the inflammation site, which may be due to its role in IFN-I signaling rather than AHR signaling.

## 1. Introduction

Post-transcriptional modifications, such as phosphorylation, ubiquitination, and ADP-ribosylation, allow for the fast and efficient integration of different stimuli during an infection and are important in controlling immune responses. Poly-ADP-ribose polymerases (PARPs) constitute a family of 17 enzymes that regulate many cellular signaling pathways [1]. They function by transferring ADP-ribose (ADPr) from nicotinamide adenine dinucleotide (NAD^+^) onto either themselves or other protein substrates, releasing nicotinamide (NAM). Most PARPs transfer one molecule of ADP-ribose (mono-ADP-ribosylation; MARylation) rather than several ADP-ribose units (poly-ADP-ribosylation; PARylation) onto their protein substrates [1,2,3]. ADP-ribosylation is involved in numerous cellular processes, including metabolism, DNA repair, protein stability, gene regulation, inflammation, and immune cell function [4].

2,3,7,8-tetrachlorodibenzo-*p*-dioxin (TCDD)-inducible poly-ADP-ribose polymerase (TIPARP), which we refer to as poly-ADP-ribose polymerase 7 (PARP7) [5], is a mono-ADP-ribosyltransferase that regulates innate immunity by repressing type I interferon signaling [6]. PARP7 also regulates stem cell pluripotency, autophagy, and gene expression [7,8,9]. PARP7 is a CCCH-zinc finger domain containing PARP with high evolutionary conservation with PARP12 and PARP13, the latter of which is also mono-ADP-ribosylated by PARP7 [1,4,10]. PARP7 expression is induced by platelet-derived growth factors [11], viral infection [12,13], nuclear hormone receptors [14,15], hypoxia [16], and aryl hydrocarbon receptor (AHR) [17]. In the latter case, PARP7 in turn represses AHR signaling through a negative feedback loop that is dependent on its catalytic activity [9,18]. Thus, decreases in PARP7 levels or inhibition of its catalytic activity results in increased AHR signaling [9,10,18]. In support of these findings, both PARP7 knockout mice and mutant mice harboring a catalytically deficient PARP7 show increased AHR activity and increased sensitivity to toxicities produced by the AHR ligand TCDD [19,20,21].

Inflammatory bowel disease (IBD) describes a group of diseases of the gastrointestinal tract, of which ulcerative colitis (UC) and Crohn’s disease are the major forms [22]. The causes of IBD are unknown, but they typically involve genetic factors, immune-mediated injury, and a dysregulation of the intestinal microbiota with a dominance of potential pathogenic bacteria [23]. Although AHR has been mostly studied for its role in chemical toxicity, it is now recognized to be an essential gatekeeper that integrates dietary, environmental, microbial, and endogenous ligand signals to modulate immune cell homeostasis and inflammation [24,25]. More than 400 AHR ligands have been identified, including the tryptophan metabolite kynurenine (KYN), the endogenous photodegradation product 6-formylindolo(3,2-b)carbazole (FICZ), and dietary ligands, such as indole-3-carbinol and one of its acid condensation products, 3,3′-diindolylmethane (DIM) [26,27,28]. Upon ligand binding, AHR enters the nucleus where it heterodimerizes with AHR nuclear translocator (ARNT). This complex binds to AHR response elements in the regulatory regions of its target genes, including the drug-metabolizing enzymes cytochrome P450 1A1 (*CYP1A1*) and *CYP1B1*, *PARP7*, cytokines, growth factors, and cell cycle regulators [29]. AHR activation significantly improves, while its loss exacerbates, dextran sodium sulphate (DSS)-induced colitis, a model of IBD [30,31,32]. This is due to the loss of anti-inflammatory intestinal type 3 innate lymphoid (ILC3) cells, decreased barrier integrity, and dysregulated intestinal inflammation [28,30]. Moreover, immune cells isolated from patients suffering from Crohn’s disease have reduced levels of AHR [31].

Emerging studies also support a critical role for PARP7 in innate immune signaling, due to its ability to negatively regulate type I interferon (IFN-I) responses [6,12]. In addition to the central role that IFN-I signalling plays in innate and antibacterial immunity, IFN-I signalling is important in the maintenance of intestinal homeostasis and suppression of intestinal inflammation by regulating the production of anti-inflammatory cytokines [33]. IFN-I signalling suppresses enteric viral and intestinal bacterial infections, but it also reduces the severity of DSS-induced colitis in mice and inhibits pro-inflammatory cytokine production in tissues isolated from patients suffering from UC [34,35].

Since PARP7 is a negative regulator of AHR and IFN-I, and loss of PARP7 expression increases both AHR and IFN-I signaling, we used a DSS-induced colitis model to investigate the effect of PARP7 loss on DSS-induced intestinal inflammation. We observed that DSS-exposed *Parp7^−/−^* mice exhibit reduced severity of disease symptoms. Our findings provide the first evidence that loss of PARP7 reduces intestinal inflammation in the DSS model of colitis. Although the mechanisms are unknown, our findings suggest that PARP7 may be a potential new therapeutic target for IBD.

## 2. Results

### 2.1. Increased AHR-Regulated CYP1A1 Levels in Hepatocytes or MEFs Isolated from Parp7^−/−^ Mice

**Figure 1:**
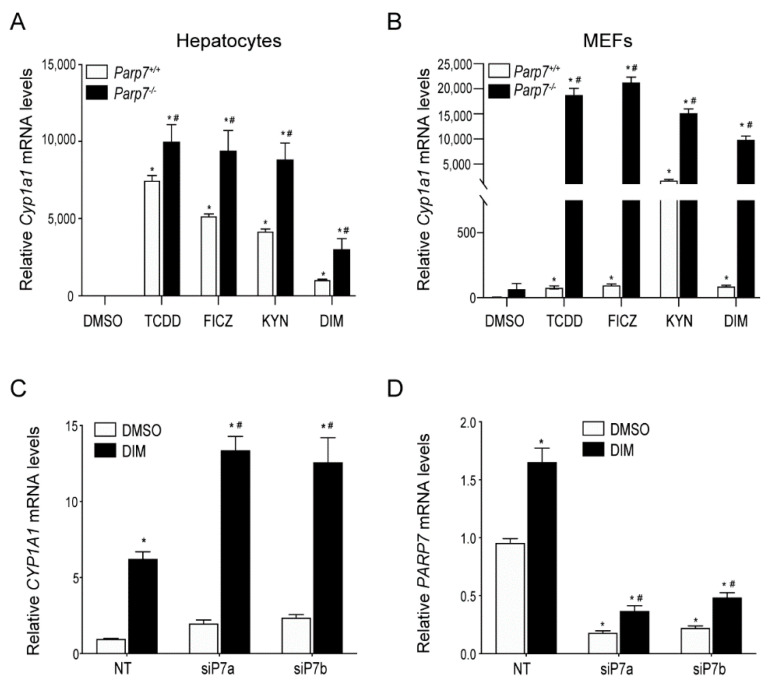
PARP7 represses *CYP1A1* expression in MEFs, hepatocytes and Caco-2 cells. AHR ligand induced *Cyp1a1* levels are higher in (**A**) hepatocytes and (**B**) MEFs isolated from *Parp7^−/−^* compared to *Parp7^+/+^* mice. Hepatocytes and MEFs were isolated from livers of *Parp7^−/−^* and *Parp7^+/+^* mice and treated with DMSO, 10 nM TCDD, 10 nM FICZ, 200 μM KYN or 10 μM DIM for 6 h. The *Cyp1a1* mRNA levels were determined with RT-qPCR. * *p* < 0.05 two-way ANOVA compared with genotyped-matched control treated hepatocytes or MEFs. ^#^ *p* < 0.05 two-way ANOVA compared with ligand matched *Parp7^+/+^* hepatocytes or MEFs. (**C**,**D**) RNAi-mediated knockdown of PARP7 increased DIM-induced *CYP1A1* levels. Caco2 cells were transfected with two different siRNAs targeting *PARP7* and treated with DMSO and 10 μM DIM for 6 h. Relative *CYP1A1* mRNA and *PARP7* mRNA levels were determined with RT-qPCR. * *p* < 0.05 two-way ANOVA compared with control treated non-targeted (NT) Caco-2 cells. ^#^ *p* < 0.05 two-way ANOVA compared with DIM-treated NT Caco-2 cells.

PARP7 functions as part of a negative feedback loop that regulates AHR signaling and protects against TCDD toxicity [9,19]. However, it is not known whether endogenous or dietary AHR ligands also increase AHR signaling in the absence of PARP7 expression. To test this, we treated hepatocytes or MEFs isolated from *Parp7^+/+^* or *Parp7^−/−^* mice with TCDD, the endogenous ligands FICZ and KYN, or DIM for 6 h and measured *Cyp1a1* mRNA levels. As observed with TCDD, the treatment of hepatocytes or MEFs isolated from *Parp7^−/−^* mice with FICZ, KYN, or DIM resulted in significantly higher *Cyp1a1* mRNA levels compared with similarly treated cells isolated from *Parp7^+/+^* mice (Figure 1A,B). We next examined whether PARP7 knockdown would result in increased AHR signaling in Caco2 cells, a human colorectal adenocarcinoma cell line. Transfection with two different siRNAs targeting PARP7 resulted in an approximately two-fold increase in *Cyp1a1* mRNA levels compared with non-targeted (NT) control transfected cells after treatment with DIM (Figure 1C), and an 80% knockdown of *Parp7* mRNA levels (Figure 1D). This level of PARP7 knockdown is consistent with what we have reported in other cell lines [9,36]. These data support PARP7’s role as a negative regulator of AHR activity, and further show that the increased AHR signaling observed after the loss or knockdown of PARP7 occurs with multiple AHR ligands across different cell lines.

### 2.2. Reduced Sensitivity of Parp7^−/−^ Mice to DSS-Induced Colitis

**Figure 2:**
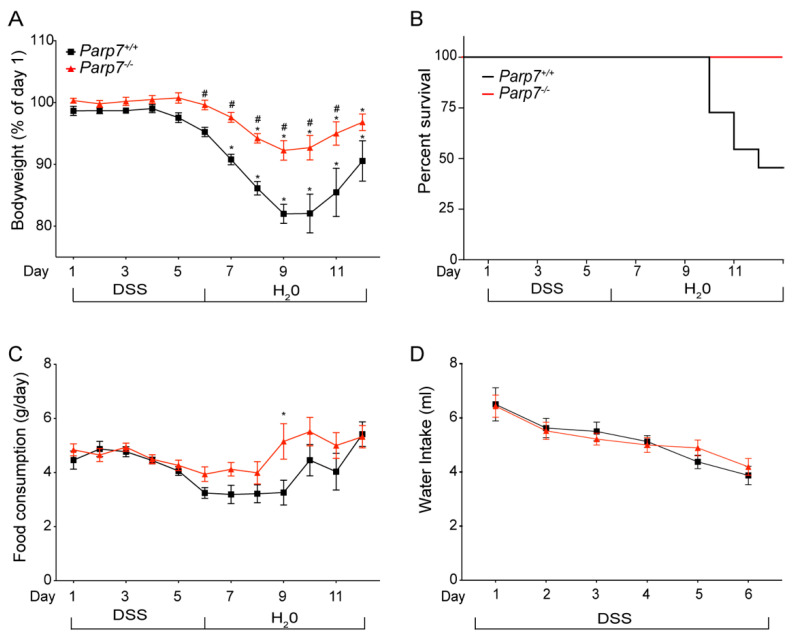
*Parp7^−/−^* mice have reduced sensitivity to DSS-induced colitis. (**A**) *Parp7^−/−^* mice exhibit less weight loss than *Parp7^+/+^* mice after exposure to 2% DSS in their drinking water. (**B**) Kaplan-Meier survival curves for *Parp7^−/−^* and *Parp7^+/+^* mice after exposure to 2% DSS in their drinking water. (**C**) Food consumption and (**D**) water intake of *Parp7^−/−^* and *Parp7^+/+^* mice at the indicated days. * *p* < 0.05 two-way ANOVA compared with day 1 genotype-matched mice. ^#^ *p* < 0.05 two-way ANOVA compared with time matched *Parp7^+/+^* mice.

AHR plays an important role in intestinal homeostasis, barrier integrity, and intestinal immunity [37]. Since the loss of PARP7 expression increases AHR signaling, we hypothesized that *Parp7^−/−^* mice would be less sensitive to the negative impacts associated with colitis and intestinal inflammation. To this end, we exposed *Parp7^+/+^* and *Parp7^−/−^* mice to the DSS-induced model of colitis. Mice were exposed to 2% DSS in their drinking water from day 0 to day 6, at which time the DSS-containing water was replaced with normal water. The mice were monitored for an additional six days until day 12. As expected, DSS treatment caused a significant loss in body weight in both genotypes starting on day 7 and continuing through day 9, before increasing again from day 10 to day 12 (Figure 2A). By day 12, there was no significant difference in body weight compared with day 1 in *Parp7^−/−^* mice. Six *Parp7^+/+^* mice did not survive, while all *Parp7^−/−^* mice survived the 13-day experiment (Figure 2B). The six DSS-treated *Parp7^+/+^* mice appeared weakened after nine days and became moribund between days 10 and 12 before being humanely euthanized. No differences in food or water intake were observed (Figure 2C,D), suggesting that the body weight loss differences between the genotypes were not due to different exposures to DSS.

**Figure 3:**
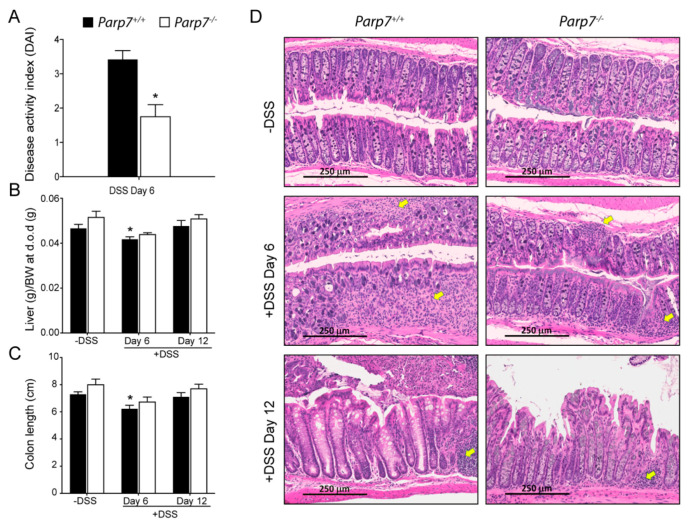
Reduced colitis symptoms in *Parp7^−/−^* mice. (**A**) *Parp7^−/^*^−^ mice present lower intestinal inflammation symptoms than *Parp7^+/+^* mice as indicated by a reduced disease activity index (DAI) on day 6 of DSS treatment. * *p* < 0.05 student’s t-test. *Parp7^+/+^* mice have a greater reduction in colon length (**B**) and liver weight (**C**) on day 6 of DSS treatment compared with *Parp7^−/^*^−^ mice. * *p* < 0.05 two-way ANOVA compared with genotype-matched and DSS treated mice. (**D**) *Parp7^−/−^* mice exhibit less tissue damage and immune cell infiltration than *Parp7^+/+^* mice. Representative H&E staining of colon section after 6 days of exposure to 2% DSS in drinking water and after water recovery. The yellow arrows indicate immune cell infiltration.

DSS administered in the drinking water disrupts the intestinal lining primarily at the site of the colon. This can lead to symptoms in mice, such as rectal prolapse, rectal bleeding, diarrhea, and hematochezia. Together, these are combined and scored as a disease activity index (DAI), which is an indication of severity of the colitis [38]. *Parp7^+/+^* mice displayed a significantly greater DAI than *Parp7^−/−^* mice on day 6 of DSS treatment, suggesting that *Parp7^−/−^* mice were less susceptible to DSS-induced pathological changes in the colon (Figure 3A). Both body weight-adjusted liver weight and colon length were more reduced in *Parp7^+/+^* than in *Parp7^−/−^* mice on day 6 (Figure 3B,C). However, no differences were observed by day 12. Histological analysis of colon sections from day 6 and day 12 DSS-treated *Parp7^+/+^* mice displayed prototypical features of DSS-induced colitis, including regions of major crypt loss and mucosal inflammation with neutrophilic infiltration (Figure 3D). In contrast, *Parp7^−/−^* mice displayed less crypt damage and inflammatory infiltration in mucosal regions at day 6. Vehicle-treated mice from both genotypes displayed normal colon mucosal morphology. Although the mice recovered in terms of body weight by day 12 (six days after removal of DSS from drinking water), there was still evidence of inflammatory infiltration within the mucosal regions of both genotypes. Crypt regeneration was particularly evident in *Parp7^+/+^* mice, which also displayed greater crypt loss at day 6.

### 2.3. Increased Expression of Proinflammatory Genes in DSS-Treated Parp7^+/+^ Compared with Parp7^−/−^ Mice

**Figure 4:**
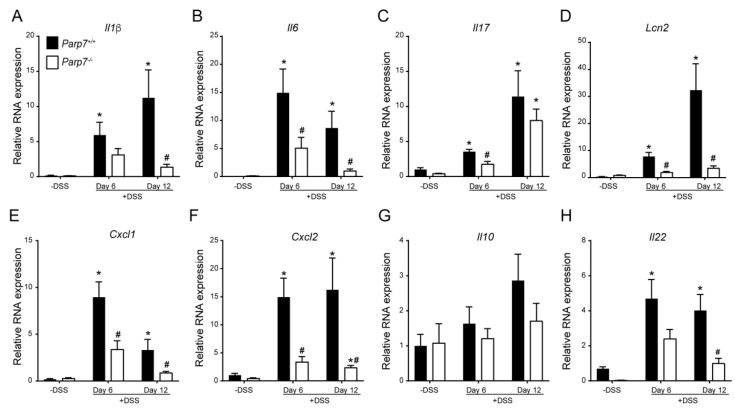
Lower expression of proinflammatory genes is observed in DSS-exposed *Parp7^−/−^* mice. Intestinal mRNA expression levels of (**A**) *Il1β*, (**B**) *Il6*, (**C**) *Il17*, (**D**) *Lcn2*, (**E**) *Cxcl1*, (**F**) *Cxcl2*, (**G**) *Il10*, and (**H**) *Il22* in colon tissue isolated from *Parp7^+/+^* and *Parp7^−/−^* mice that were not exposed to DSS (-DSS) or exposed to 2% DSS in their drinking water for 6 days or 12 days (6 days DSS followed by 6 days water). The relative mRNA levels of the indicated genes were determined with RT-qPCR. * *p* < 0.05 two-way ANOVA compared with genotyped-matched and DSS treated animals. ^#^ *p* < 0.05 two-way ANOVA compared with time matched *Parp7^+/+^* mice.

We next examined the mRNA levels of numerous proinflammatory cytokines, chemokines, and lipocalin-2 (*Lcn2*), a potential biomarker for inflammatory bowel disease. The levels of *Il-1β*, *Il-6*, *Il-17,* and *Lcn2* were significantly increased in colon tissue from DSS-treated *Parp7^+/+^* compared with *Parp7^−/−^* mice on day 6, day 12, or both (Figure 4A–D). The levels of *Cxcl1* and *Cxcl2* were also significantly lower in colons of *Parp7^−/−^* compared with *Parp7^+/+^* mice (Figure 4E,F). No significant differences in the levels of the anti-inflammatory cytokine *Il10* were observed between genotypes (Figure 4G). *Il-22* levels were significantly increased in *Parp7^+/+^* mice compared with *Parp7^−/−^* mice. However, this was only observed on day 12 (Figure 4H). These data demonstrate that PARP7 loss resulted in reduced DSS-induced intestinal inflammation.

### 2.4. No Changes in Cyp1a1 and Cyp1b1 Expression Levels in DSS-Treated Parp7^+/+^ Compared with Parp7^−/−^ Mice

**Figure 5:**
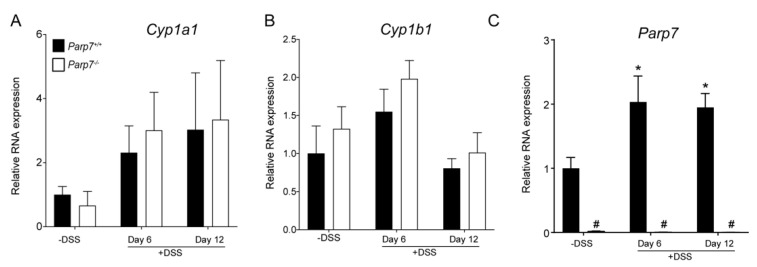
*Parp7*, but not *Cyp1a1* and *Cyp1b1* expression levels are increased in response to DSS. The expression levels of (**A**) *Cyp1a1* and (**B**) *Cyp1b1* in colonic tissue are unaffected by genotype or exposure to 2% DSS in drinking water. (**C**) *Parp7* expression is induced in colon tissue isolated from DSS treated *Parp7^+/+^* mice. * *p* < 0.05 two-way ANOVA compared with genotyped-matched and DSS treated animals. ^#^ *p* < 0.05 two-way ANOVA compared with time matched *Parp7^+/+^* mice.

Increased AHR signaling by endogenous, microbial produced or dietary ligand activation protects against DSS-induced inflammation and toxicity [28,37]. Since PARP7 functions as a negative regulator of AHR signaling, we next examined the colonic mRNA expression levels of the AHR target genes *Cyp1a1*, *Cyp1b1,* and *Parp7*. No significant differences in *Cyp1a1* or *Cyp1b1* mRNA levels were observed for either genotype (Figure 5A,B). *Parp7* mRNA levels were, however, significantly increased in response to DSS at day 6, and remained significantly elevated at day 12 in *Parp7^+/+^* mice. No *Parp7* mRNA was detected in *Parp7^−/−^* mice (Figure 5C).

### 2.5. Influence of PARP7 Loss on the Composition of the Intestinal Microbiota

**Figure 6:**
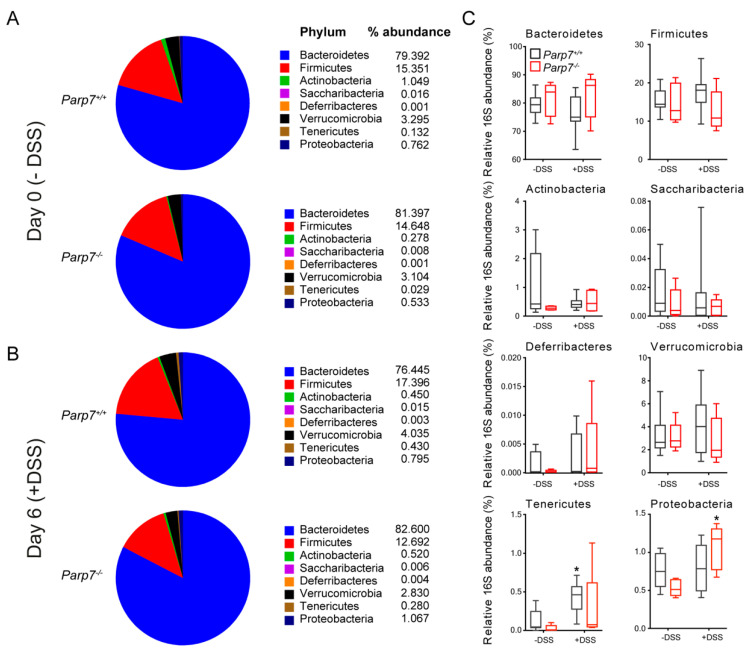
DSS perturbs the composition of intestinal microbiota differently in Parp7^−/−^ and Parp7^+/+^ mice. (**A**–**C**) Fecal pellets were collected from *Parp7^−/−^* and *Parp7^+/+^* mice (**A**) before exposure to DSS (day 0) and (**B**) after 6 days with 2% DSS in their drinking water. The proportion of different bacterial phyla was quantified by RT-qPCR using the 16S RNA as described in Materials and Methods. * *p* < 0.05 two-way ANOVA compared with genotyped-matched and DSS treated animals.

We next analyzed the microbial composition at the phyla level using a qPCR-based approach. Bacteroidetes were found to be the most abundant of the bacterial phyla, followed by Firmicutes in both genotypes on day 0 and day 6 (Figure 6A,B). We did not observe any significant differences in microbial composition between genotypes (Figure 6C). However, DSS administration resulted in a significant increase in Tenericutes in *Parp7^+/+^* and Proteobacteria in *Parp7^−/−^* mice, respectively.

## 3. Discussion

AHR and IFN-I are important regulators of intestinal homeostasis, barrier function, and inflammation [33,37]. Because PARP7 negatively regulates AHR and IFN-I signaling [6,9], we hypothesized that the loss of PARP7 expression would improve therapeutic outcomes in the DSS model of colitis. We observed reduced severity of disease and lower levels of many pro-inflammatory cytokines and chemokines in *Parp7^−/−^* compared with the *Parp7^+/+^* mice. However, we did not detect increased intestinal expression of AHR target genes in *Parp7^−/−^* mice, suggesting that the reduced inflammation may be due to PARP7’s role in IFN-I or other inflammatory signaling pathways. Although the negative effects of DSS-induced colitis were reduced but not completely prevented, our findings suggest that inhibiting PARP7 could have a beneficial role in treating colitis.

Current data support a critical role for AHR in intestinal homeostasis, inflammation, immunity, and barrier integrity [37,39]. Dietary AHR ligands promote maintenance of intraepithelial T cells (IELs, e.g., γδ T cells and CD8αα T cells), as well as innate lymphoid cells (ILCs) [37]. Treatment with the natural AHR ligand, Indigo Naturalis, for eight weeks resulted in effective clinical responses in patients with ulcerative colitis [40]. A previous study reported that DSS increased CYP1A1 mRNA levels in C57BL/6 wild-type mice, suggesting that endogenous AHR ligands are induced because of the intestinal inflammation [41]. We did not observe significant increases in CYP1A1 levels in *Parp7^+/+^* or in *Parp7^−/−^* mice. This was surprising, since PARP7 loss or knockdown increases AHR regulated target genes in different cell lines and mice exposed to TCDD [19,20,36,42]. PARP7 levels were, however, significantly increased in DSS-treated *Parp7^+/+^* mice, supporting the notion that inflammation induces endogenous ligands that activate AHR to maintain intestinal homeostasis [41]. Loss of AHR or diets devoid of AHR ligands increase the susceptibility to acute intestinal inflammation in models of colitis. Although much of the protective role of AHR in the gut has been attributed to its activation by dietary and/or microbiota derived ligands, TCDD also ameliorates DSS- and 2,4,6-trinitrobenzenesulfonic acid (TNBS)-induced colitis by reducing inflammation and promoting regulatory immune cells [43,44]. However, resveratrol (3,4,5-trihydroxy-trans-stilbene), an AHR antagonist and naturally occurring compound found in the skin of red grapes and in red wine, suppresses inflammation and colon cancer associated with colitis in rodents [45,46,47]. Whether resveratrol’s suppression of colitis is independent of AHR and rather due to its more general antioxidant and anti-inflammatory properties has not been experimentally determined. AHR signaling is regulated through the increased catalytic degradation of AHR-activating ligand via the induction of cytochrome P450 family 1 (CYP1) enzymes, through transcriptional repression via AHR repressor (AHRR), and via ADP-ribosylation, transrepression, and proteolytic degradation by PARP7 [19]. Transgenic *Cyp1a1* mice and the specific overexpression of CYP1A1 in mouse IECs are sufficient to phenocopy the inability of *Ahr^−/−^* mice to recover from enteric infection and repair intestinal barrier integrity [48], supporting the central role of AHR and AHR ligand availability in intestinal homeostasis.

*Cyp1a1* expression is elevated with loss of AHRR expression in a tissue-specific manner in *Ahrr*- knockout mice [49]. Recent studies reveal that *Ahrr* deletion in mice prevents excessive proinflammatory signaling upon LPS challenge but aggravates symptoms of DSS induced colitis [50]. This suggests that AHRR exhibits context-specific proinflammatory and anti-inflammatory functions, and its role in intestinal inflammation may be distinct from its ability to repress AHR. In contrast, here, we show that the loss of PARP7, which also functions as an AHR repressor, reduced inflammation and improved symptoms of DSS colitis. However, we saw no significant differences in AHR target gene expression in colon tissue isolated from *Parp7^−/−^* compared with *Parp7^+/+^* mice. Little is known about PARP7’s role or its protein levels in intestinal epithelium or in intestinal immune cells. We cannot rule out the possibility that PARP7 deficiency enhances the beneficial actions of AHR in resident or recruited intestinal immune cells in a cell-type specific manner to promote an anti-inflammatory environment. Future studies will be necessary to characterize PARP7’s function in the gut and in intestinal associated immune cells using cell- and tissue-specific deficient mouse models or through chimeric immune cell transfer studies, to better define its role in intestinal health and inflammation.

IFN signaling is induced by nucleic and non-nucleic acids known as damage-associated molecular patterns, (DAMPs). DAMPs are released upon viral infection, endogenous signaling, and cell damage and are recognized by pattern recognition receptors. PARP7 plays a critical role in innate immunity and IFN-I signaling [6]. IFN-I signaling reduces intestinal inflammation and the interferon-α/β receptor (IFNAR), an IFN-I receptor, is a susceptible region for human IBD [51]. IFN-I signaling protects in DSS-induced colitis models through suppression of pro-inflammatory cytokine production, including IL1β [52]. IFN-I protects against viral and bacterial intestinal infections through both the innate and adaptive immune responses, it enhances barrier function, and it prevents dysbiosis [33]. PARP7 deficiency increases IFN-I signaling in response to viral infection by preventing TBK1-dependent suppression of the IFN-I activation cascade [6]. Interferon regulatory factor 3 (IRF3), IRF7, and IRF9 regulate the production of IFN-I. IRF3 deficient mice exhibit severe colitis, showing that dysregulation of IRF/IFN-I signaling is involved in the pathogenesis of IBD [53]. Thus, the reduction in DSS-induced intestinal inflammation observed in *Parp7^−/−^* mice might be related to PARP7’s impact on interferon expression and not on the regulation of AHR activity. We propose that DAMPs derived from gut microbiota activate IFN-I to reduce inflammation and maintain intestinal homeostasis. In the absence of PARP7, this IFN-I-dependent protection would be enhanced. However, persistent levels of IFN-I could over time lead to enhanced chemokine and cytokine production, which may worsen the progression of colitis over time. Other studies have reported that increased IFN-I signaling resulted in delayed recovery from intestinal inflammation due to increased chemokine production and infiltration of neutrophils and inflammatory monocytes [54]. These opposing roles of IFN-I signaling during disease progression may explain the varying effects of IFN-I treatment for IBD patients [55]. Targeting PARP7 as opposed to directly targeting IFN-I may be a potential alternative strategy, since a delay in recovery was not observed in DSS-exposed *Parp7^−/−^* mice.

Increased AHR and IFN-I signaling is also phenocopied by a loss of PARP7’s catalytic activity through the introduction of a single histidine to alanine mutation at amino acid 532 of the protein [6,9,21]. Recently, two small molecule inhibitors of PARP7 have been reported [10,56]. One of the inhibitors, known as RBN-2397, is currently in a Phase 1 clinical trial for patients with advanced-stage solid tumors (NCT04053673). The trial exploits RBN-2397’s inhibition of PARP7 to cause increased IFN-I dependent activated immune cell mediated killing [57]. A deficiency in PARP1, the founding member of the PARP family, or pretreatment with pan-PARP inhibitors also improves symptoms in experimental colitis [23,58]. These findings suggest that PARP inhibitors with selectivity for PARP1 or PARP7 may be suitable in the treatment of ulcerative colitis and other chronic intestinal inflammation disorders.

The gut microbiome is often altered to a less diverse and less beneficial composition in IBD. The causes of this dysbiosis, and whether it is necessary for the development of the inflammation, remains unclear. In patients with IBD, Proteobacteria are increased, while Firmicutes are often decreased in both amount and in diversity [59]. Proteobacteria contain known pathogenic and non-pathogenic strains of bacteria. The increase in Proteobacteria observed in DSS-exposed *Parp7^−/−^* mice in our study is consistent with human IBD studies [59], but it is unclear whether and how this contributes to the amelioration of intestinal inflammation. Bacteroidetes was the most abundant of the bacterial phyla, followed by Firmicutes in both genotypes on day 0 and day 6 of DSS treatment, which agrees with other studies using C57BL/6 mice [60,61]. The Firmicutes/Bacteroidetes (F/B) ratio has been reported to maintain normal intestinal homeostasis and an altered ratio is observed in some diseases [62]. An increased F/B ratio is associated with obesity, while a decreased F/B ratio is associated with IBD [62]. Many Firmicutes produce the short chain fatty acid (SCFA) butyrate upon fermentation of indigestible carbohydrates. Other SCFAs, mainly propionate and acetate, are produced mainly by Bacteroidetes. Butyrate and other SCFAs link host nutrition to intestinal homeostasis, barrier functions, and immune modulation [63]. Butyrate was also recently reported to induce the transcription of AHR target genes in IECs [64]. Despite the reduction in intestinal inflammation in DSS exposed *Parp7^−/−^* compared with *Parp7^+/+^* mice, we did not observe any significant differences in the composition of microbiota at the phylum level, nor did we observe a change in the F/B ratio. Metagenomic analyses of the microbiomes will be needed to determine differences in microbial communities between DSS-treated *Parp7^+/+^* and *Parp7^−/−^* mice.

In summary, we provide the first evidence that the loss of PARP7 reduces intestinal inflammation in a model of colitis. Although the mechanisms are unknown, our data are consistent with an inhibitory function of PARP7 on AHR signaling and IFN-I signaling. Our findings also suggest that the inhibition of PARP7 alone or in combination with diets rich in AHR ligands may provide a therapeutic benefit for patients with inflammatory intestinal disorders.

## 4. Materials and Methods

### 4.1. Chemicals

Dextran sulfate sodium (DSS) salt reagent grade from MP Biomedicals (Irvine, CA, USA) was dissolved to a concentration of 2% in autoclaved drinking water. The 2,3,7,8-tetrachlorodibenzo-*p*-dioxin (TCDD) was purchased from Accustandard (New Haven, CT, USA). Dimethyl sulfoxide (DMSO), 3,3′-diindolylmethane (DIM), kynurenine (KYN), FICZ (6-formylindolo(3,2-b)carbazole), and all other chemicals and biological reagents were purchased from Merck (Frankfurt, Germany) unless stated otherwise.

### 4.2. Animals and In Vivo Dextran Sulfate Sodium (DSS) Treatment

For all studies, 8-week-old male *Parp7^+/+^* and *Parp7^−/−^* mice were used. The generation and characterization of these *Parp7^−/−^* mice, which were referred to as *Tiparp^tm1d/tm1d^* and *Tiparp^Ex3−/−^* mice, have been described previously [20]. The mice were placed in single cages and given either normal drinking water or water containing 2% (*w*/*v*) DSS for 6 days to induce inflammatory bowel disease (IBD) [65]. In some experiments, the DSS-containing water was replaced with regular water after 6 days and the mice were monitored until day 12, while in other studies animals were euthanized on day 6. Water and food consumption were measured daily. Body weight and signs of IBD were monitored daily, including diarrhea, weight loss, dehydration, hematochezia, weakness, and rectal prolapse. At endpoint, the animals were euthanized by cervical dislocation, and liver, intestines, and colon were removed for gene expression, histological, and biochemical analyses. Feces were collected on days 0, 6, and 12 and used to evaluate changes in the microbiome using real-time qPCR-based methods as described by others [66]. All animals were bred and cared for at the University of Toronto. Care and treatment of animals followed the guidelines set by the Canadian Council on Animal Care and were approved by the University of Toronto Animal Care Committee.

### 4.3. Histology

Hematoxylin & eosin staining was performed using standard methods with representative images provided. Formalin-fixed tissues were provided to the HistoCore Facility at the Princess Margaret Cancer Centre, Toronto, Ontario for histology sample processing, staining, and scanning of stained slides.

### 4.4. LiCl Clean-Up Protocol for DNA and RNA

DSS is a known inhibitor of PCR. To minimize the carry-over of DSS during DNA and RNA purification, a LiCl precipitation specific for DNA or RNA was performed as described previously [66]. For the DNA, a 1:1 ratio of 5 M LiCl was added to the diluted DNA and placed on ice for one hour. The solution was then centrifuged for 10 min at 4 °C. The supernatant was removed by pipetting and the DNA was re-suspended in 100 µL of LiCl and left on ice for another 15 min before centrifuging for 10 min at 4 °C. After spinning, the supernatant was discarded, and the DNA pellet was re-suspended in 100 µL molecular grade water. The DNA was precipitated and centrifuged for 10 min at 20,000× *g*. The pellet was washed with 500 µL 70% EtOH, centrifuged and air-dried at 60 °C for 20 min, and resuspended in 50 µL molecular grade water. For the isolation of RNA, a similar procedure was followed, except that a 0.1:1 ratio of 5M LiCl to RNA was used in the first step of the purification.

### 4.5. Hepatocyte and Mouse Embryonic Fibroblast (MEF) Isolation

*Parp7^+/+^* or *Parp7^−/−^* male mice (8–10 weeks old) were used to isolate primary hepatocytes. Mouse liver was perfused with liver perfusion medium (Merck, Frankfurt, Germany) for 10 min followed by liver digestion medium for 10 min. Freshly prepared hepatocytes were seeded at a final density of 5 × 10^5^ cells/well onto type I collagen coated six-well plates in attachment medium (William’s E media, 10% dextran-coated charcoal (DCC) stripped fetal bovine serum (FBS), 1× penicillin/streptomycin, and 10 nM insulin). The medium was changed 2 h after plating, and all experiments were performed on the second day. Ligands (TCDD, FICZ, KYN, DIM) were added to the cells in M199 media with 5% DCC-FBS and cells were harvested 6 h after ligand treatment for RNA extraction. Isolation and immortalization of *Parp7^+/+^* or *Parp7^−/−^* mouse embryonic fibroblasts (MEFs) were performed as described previously [9].

### 4.6. RNAi Knockdown Studies

Caco-2 cells (American Tissue Culture Collection [ATCC] HTB-37) were grown in Dulbecco’s Modified Eagle Medium (DMEM) (Merck, Frankfurt, Germany) supplemented with 20% FBS and 2 mM L-glutamine and maintained at 37 °C in a humidified environment with 5% CO_2_. For RNAi-knockdown experiments, Caco-2 cells were transfected with 30 nM of non-target siRNA (NT2; Dharmacon (Lafayette, CO, USA); catalog no D-001206-14), or two siRNAs targeting PARP7, referred to as siRNA P7a (siP7a) (Thermo Fisher Scientific; s24857) and siRNA P7b (siP7b) (Thermo Fisher Scientific (Middletown, VA, USA); s24858), using RNAiMAX according to the manufacturer’s instructions (Thermo Fisher Scientific). Twenty-four hours post transfection, cells were treated with DMSO (0.01%) or 10 μM DIM for 6 h.

### 4.7. RNA Extraction and Gene Expression Analysis

After ligand treatment, total RNA was isolated from hepatocytes, MEFs, or Caco2 cells using the Aurum Total RNA mini kit according to the manufacturer’s instructions (Bio-Rad, Hercules, CA, USA). Tissue from the distal end of the colon was dissected, fecal matter pushed out, and the inside and outside flushed with PBS. The samples were flash frozen in liquid nitrogen and stored at −80 °C. Colon tissue was homogenized in 500 µL TRizol (Thermo Fisher Scientific). After extraction with 100 µL chloroform and centrifugation, the upper layer was transferred to a new tube containing 300 µL of 70% ethanol. After mixing, the RNA containing solution was added to Aurum RNA purification columns, and the RNA was purified according to the manufacturer’s instructions. The purified RNA was diluted to 50 ng/µL, and 10 µL were used in a High-Capacity cDNA Reverse Transcription Kit reaction according to the manufacturer’s instructions (Applied Biosystems, Waltham, MA, USA). PCR Primers used to amplify target transcripts are described elsewhere [19]. All genes were normalized to TATA binding protein (*Tbp*) RNA levels and analyzed using the comparative C_T_ (ΔΔ C_T_) method.

### 4.8. DNA Isolation and Real Time-qPCR Analysis

DNA was extracted from frozen stool samples using QIAamp DNA Stool Mini Kit (QIAGEN, Hilden, Germany) according to the manufacturer’s protocol with the following modifications. Feces were thawed in lysis buffer at 37 °C at intervals of 5 min with 1 min of vortex on full speed. This was repeated 3–5 times to ensure that the fecal samples were fully solubilized. To increase the yield of DNA, 600–900 μL of supernatant was loaded and passed through the filter in the spin column prior to the LiCl clean-up protocol. Differences in the levels of bacteria at the phylum level were determined from fecal DNA by qPCR using PCR primers described previously [61]. Each reaction consisted of 5 µL SYBR green, 1 µL DNA with a concentration of 5 ng/μL, 0.3 µL each of forward and reverse primer with a concentration of 10 nM, and 3.4 µL molecular grade H_2_O to a final volume of 10 μL.

### 4.9. Statistical Analysis

Statistical analysis for significance (*p* < 0.05) was determined using the Student’s *t*-test or two-way ANOVA using GraphPad Prism 8.0 (GraphPad Software, San Diego, CA, USA). Differences were noted as significant * *p* < 0.05 or ** *p* < 0.01, unless specified otherwise.
